# Study on plasma metabolomics profiling of depression in Chinese community-dwelling older adults based on untargeted LC/GC‒MS

**DOI:** 10.1038/s41598-024-60836-7

**Published:** 2024-05-05

**Authors:** Jiangling Guo, Peipei Han, Yaqing Zheng, Yahui Wu, Kai Zheng, Chuanjun Huang, Yue Wang, Cheng Chen, Yiqiong Qi, Xiaoyu Chen, Qiongying Tao, Jiayi Zhai, Qi Guo

**Affiliations:** 1 Graduate School, Shanghai University of Traditional Chinese Medicine, Shanghai, China; 2 Department of Rehabilitation Medicine, Shanghai University of Medicine and Health Sciences Affiliated Zhoupu Hospital, 279 Zhouzhu Highway, Pudong New Area, Shanghai, 201318 China; 3 Fujian Provincial Hospital, Fujian, China; 4 School of Health Science and Engineering, University of Shanghai for Science and Technology, Shanghai, China; 5 School of Health, Fujian Medical University, Fuzhou, Fujian China; 6 Department of Sport Rehabilitation, Shanghai University of Sport, Shanghai, China; 7 Jiading Subdistrict Community Health Center, Shanghai, China; 8 Shanghai University of Traditional Chinese Medicine, Shanghai, China

**Keywords:** Depression, Nontargeted metabolomics, GC/LC‒MS, Receiver operating characteristic curve, Metabolic pathways, Biochemistry, Computational biology and bioinformatics, Molecular biology, Neuroscience

## Abstract

Depression is a serious psychiatric illness that causes great inconvenience to the lives of elderly individuals. However, the diagnosis of depression is somewhat subjective. Nontargeted gas chromatography (GC)/liquid chromatography (LC)–mass spectrometry (MS) was used to study the plasma metabolic profile and identify objective markers for depression and metabolic pathway variation. We recruited 379 Chinese community-dwelling individuals aged ≥ 65. Plasma samples were collected and detected by GC/LC‒MS. Orthogonal partial least squares discriminant analysis and a heatmap were utilized to distinguish the metabolites. Receiver operating characteristic curves were constructed to evaluate the diagnostic value of these differential metabolites. Additionally, metabolic pathway enrichment was performed to reveal metabolic pathway variation. According to our standard, 49 people were included in the depression cohort (DC), and 49 people age- and sex-matched individuals were included in the non-depression cohort (NDC). 64 metabolites identified via GC‒MS and 73 metabolites identified via LC‒MS had significant contributions to the differentiation between the DC and NDC, with VIP values > 1 and *p* values < 0.05. Three substances were detected by both methods: hypoxanthine, phytosphingosine, and xanthine. Furthermore, 1-(sn-glycero-3-phospho)-1D-myo-inositol had the largest area under the curve (AUC) value (AUC = 0.842). The purine metabolic pathway is the most important change in metabolic pathways. These findings show that there were differences in plasma metabolites between the depression cohort and the non-depression cohort. These identified differential metabolites may be markers of depression and can be used to study the changes in depression metabolic pathways.

## Introduction

Currently, China’s population is ageing rapidly. The number of individuals over the age of 60 exceeded 264 million in 2020^1^, and the elderly population is estimated to increase to 480 million by 2050^2^. According to research, depression in elderly people is a major public health problem, with an estimated point prevalence ranging from 7.8 to 34.8% in individuals over 60 years old in Asian countries^3^. Depression is a persistent and serious psychiatric illness^4^ and places a great burden on individuals in this age group, causing cognitive impairment^5^, physical activity ability decline^6^, and fall risk increase^7^. However, there is a huge challenge with respect to the recognition and accurate diagnosis of these disorders in older adults^8^. The main reason is that the clinical diagnosis of depression is often not achieved through administering scales, which are somewhat subjective and lead to a high rate of misdiagnosis^9^. Therefore, objective measures are needed in the diagnosis of depression.

The goal of metabolomics is to conduct a comprehensive study of all substances with low molecular weights in body fluids, cells, tissues, and organs^10^. Comprehensive metabolite profiling, or “metabolomics”, defines the chemical phenotype of human subjects and animal models and, as such, has unique potential for defining biomarkers that predict disease incidence, severity, and progression and for casting new light on underlying mechanistic abnormalities^11^. Currently, there are many detection methods for metabolomics research, such as gas chromatography‒mass spectrometry (GC‒MS) and liquid chromatography–mass spectrometry (LC‒MS). A previous study analysed the plasma of children and adolescents with major depressive disorder using LC‒MS and identified polyunsaturated fatty acid metabolism, purine metabolism, and inosine as potential independent diagnostic biomarkers^12^. Moreover, in plasma samples of from young adults with depression identified via LC‒MS, branched-chain amino acids showed a significant association with depression^13^. In addition, blood samples from patients with postpartum depression were analysed via GC‒MS, and the results revealed that serine/threonine and glycerol lipid metabolism were changed^14^. However, these studies did not focus on elderly individuals and only used GC‒MS or LC‒MS rather than both methods. No single metabolomics platform could provide adequate coverage of the entire human metabolome in biosamples^15^. The combination of gas chromatography and liquid chromatography can overcome the barrier of liquid chromatography only detecting polarity, heat resistance, and nonvolatile metabolites and can also overcome the limitation of low chromatographic resolution that is associated with liquid chromatography^16,17^.

Our study analysed the changes in the plasma metabolism profile of elderly, Chinese, community-dwelling individuals with depression by GC/LC‒MS. Our goal is to help diagnose and effectively treat potential biomarkers of depression in this age group and to discover metabolic pathway alterations.

## Materials and methods

### Participants

**Figure 1:**
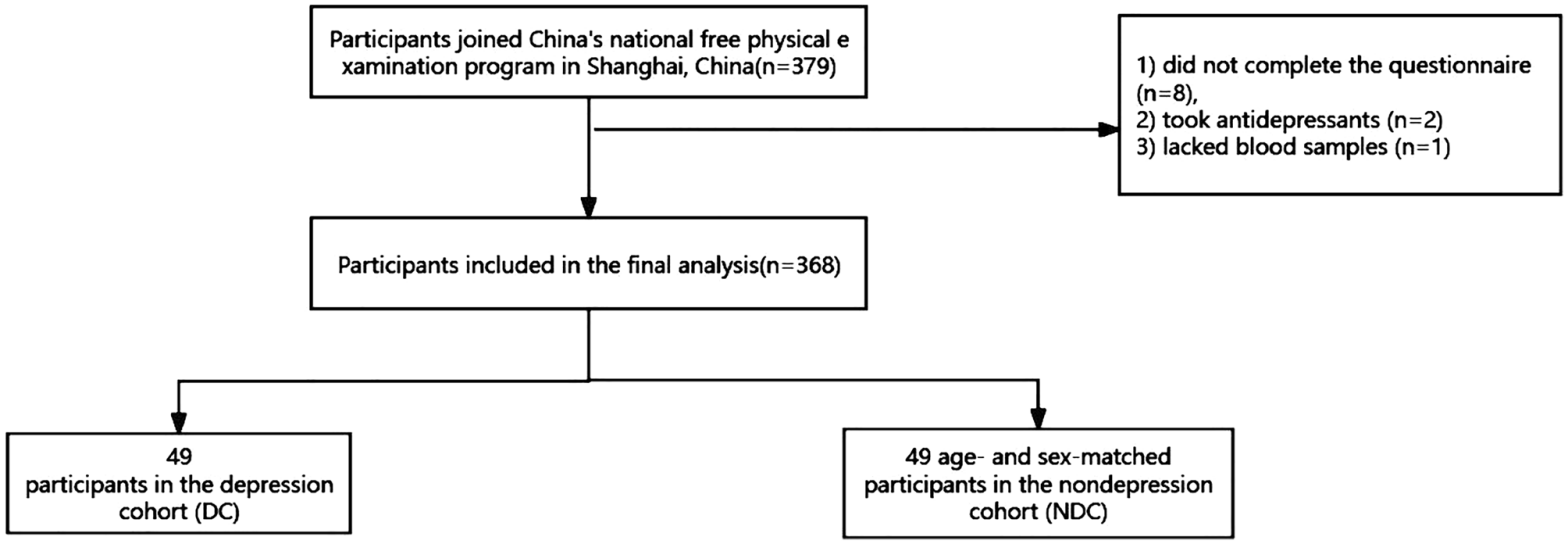
A flowchart of participant selection.

All of the subjects were individuals aged ≥ 65 who n. This study included 379 subjects who were invited to complete a comprehensive geriatric assessment and a face-to-face interview in the local community hospital. Our questionnaire assessed sociodemographic, lifestyle and health information. Sociodemographic variables included age and sex. Lifestyle includes smoking, drinking and daily activity levels. Daily activity levels were measured using the short form of the International Physical Activity Questionnaire (IPAQ)^18^. Health information included BMI, chronic conditions (such as diabetes, hypertension, hyperlipidemia, stroke, and heart disease, medication use and cognitive function. Cognitive function was assessed by the Mini-Mental State Examination (MMSE)^19^. Details of the questionnaire have been described in our previous study^20^. We excluded subjects who (1) did not complete the questionnaire (n = 8), (2) took antidepressants (n = 2) and (3) lacked blood samples (n = 1). Our subject screening process is shown in Fig. 1. The protocol of our study was reviewed and approved by the ethics committee at Shanghai University of Medicine and Health Sciences, China, and the methods were carried out in accordance with the principles of the Declaration of Helsinki. All the subjects provided informed consent before participation.

### Measures of depression

Depression was measured using the 30-item geriatric depression scale (GDS)^21^. On this scale, items 2–4, 6, 8, 10–14, 16–18, 20, 22–26, and 28 are scored 1 point if answered “yes”, and items 1, 5, 7, 9, 15, 19, 21, 27, 29 and 30 are scored 1 point if answered “no”. A total score of more than 10 points was considered to indicate depression. According to our standard, there were 49 subjects in the depression cohort (DC) and 49 age- and sex-matched individuals in the non-depression cohort (NDC).

### Sample collection and preparation

Sample collection and LC‒MS have been described in detail in our previous study^19^. Each plasma sample was collected from the study subjects on an empty stomach in the morning and stored at − 80 °C until analysis. Before LC‒MS, 150 μl of plasma that was thawed at room temperature was added to a 1.5-ml Eppendorf tube with 10 μl of 2-chlorophenylalanine (0.3 mg/ml) dissolved in methanol as an internal standard and 450 μl of a mixture of methanol/acetonitrile (2/1) to remove the protein and then vortexed for 1 min. The mixture was extracted by ultrasonication for 10 min, stored for 30 min (− 20℃) and then centrifuged at 4 °C for 10 min (13,000 rpm). Two hundred microlitres of supernatant was dried in a freeze concentration centrifuge dryer, redissolved in 300 μl of methanol/water (1/4), vortexed for 30 s, and extracted by ultrasonication for 3 min. The sample was centrifuged at 4 °C for 10 min (13,000 rpm), and 150 μl of supernatant was filtered through 0.22-μm microfilters and transferred to LC vials. The vials were stored at − 80 °C until LC‒MS.

The pretreatment for GC‒MS was similar to that for LC‒MS. A total of 150 μl of plasma was added to an Eppendorf tube with 20 μl of 2-chlorophenylalanine (0.3 mg/ml) dissolved in methanol as an internal standard and vortexed for 10 s. Then, 450 μl of an ice-cold mixture of methanol/acetonitrile (2/1, v/v) to remove the protein was added to the tube and vortexed for 30 s. The mixture was extracted by ultrasonication in an ice water bath for 10 min, stored for 30 min (− 20 °C), and centrifuged at 4 °C for 10 min (13,000 rpm). Two hundred millilitres of supernatant was placed into a new glass bottle, dried in a freeze concentration centrifuge and added to 80 μL of 15 mg/mL methoxylamine hydrochloride in pyridine. The resultant mixture was vortexed for 2 min and incubated at 37 °C for 90 min. Then, 50 μL of BSTFA (with 1% TMCS) and 20 μL of n-hexane were added into the bottle, and the bottle was vortexed violently for 2 min and derivatized at 70 °C for 60 min. The samples were placed at room temperature for 30 min before GC‒MS.

### LC‒MS and GC‒MS

LC‒MS was performed on the ACQUITY UPLC I-Class system (Waters Corporation, Miford, USA) coupled with VION IMS QT of the high-resolution mass spectrometer (Waters Corporation, Milford, USA). An ACQUITY UPLC BEH C18 column (1.7 μm, 2.1 × 100 mm) was employed in both the positive and negative models. GC‒MS was performed on an Agilent 7890B gas chromatography system coupled to an Agilent 5977A MSD system (Agilent Technologies Inc., CA, USA). A DB-5MSf used-silica capillary column (30 m × 0.25 mm × 0.25 μm, Agilent J& W Scientific, Folsom, CA, USA) was utilized to separate the derivatives. To monitor the stability and repeatability of LC‒MS and GC‒MS, QC samples were inserted regularly and analysed in every ten samples.

### Metabolite identification and analysis

The LC‒MS data were analysed using Proggenesis Qi software version 2.3 (Nonlinear, Dynamics, Newcastle, UK). First, the software is used to carry out meaningful data mining and perform advanced alignment, picking, normalization, and retention time (RT) correction. The obtained characteristic matrix includes information about the mass charge ratio (m/z), RT, and peak intensities. Then, the identification of metabolites was based on precise m/z, secondary fragments, and isotope distribution using the human metabolome database (HMDB), Human Metabolome Database (HMDB) (http://www.hmdb.ca/), lipid maps (version 2.3) (http://www.lipidmaps.org/), METLIN (http://metlin.scripps.edu/), and self-built databases (EMDB) for qualitative analysis.

The GC‒MS data used the software MS-DIAL version 2.74 for peak detection, peak identification, characterization, peak alignment, wave filtering, etc. Metabolites were annotated through the LUG database (Untargeted database of GC–MS rom Lumingbio). The raw data matrix was obtained from the raw data with a three-dimensional dataset, including sample information, the name of the peak of each substance, retention time, retention index, mass-to-charge ratio, and signal intensity, after alignment with the Statistical Compare component. The internal standards with RSD > 0.3 were used to segment and normalize all peak signal intensities in each sample, and the segmented and normalized results were removed redundancy and merged peak to obtain the data matrix.

A total of 1008 compound identifications detected by LC‒MS and 446 compound identifications detected by GC‒MS were automatically linked to the compounds. Finally, orthogonal partial least-squares discriminant analysis (OPLS-DA) was used to visualize the differences in metabolites between DC and NDC, and 200 response permutation tests (RPTs), including parameters such as R2 and Q2, were used to quantify the goodness of fit and assess the reliability of the established models. If these parameters were close to 1.0, the model was considered valid. Multidimensional coupling and single-dimensional analysis were used to select different metabolites between groups. The variable importance in projection (VIP) generated in OPLS-DA represented differential metabolites with biological significance. Furthermore, the significance of differential metabolites was further verified by Student’s t test. Variables with VIP > 1.0 and *p* < 0.05 were considered to be differential metabolites. To quantify the diagnostic performance of differential metabolites, a receiver operating characteristic curve (ROC) analysis was carried out, and the value of the area under the ROC curve (AUC) was calculated.

### Pathway analysis

To determine the mechanism of metabolic pathway variation, the differential metabolites were based on the Kyoto Encyclopedia of Genes and Genomes (KEGG) database (http://www.kegg.jp/kegg/pathway.html) to carry out metabolic pathway enrichment analysis. Their KEGG ID and pathway were found, and then the number of metabolites enriched in the corresponding pathway was calculated. The pathway with a *p* < 0.05 was selected as an enriched pathway; its calculation formula is given as follows:

$$P = \mathop \sum \limits_{i = 0}^{m - 1} \frac{{\left( \frac{M}{i} \right)\left( {\frac{N - M}{{n - i}}} \right)}}{\frac{N}{n}}$$

where N is the total number of metabolites, n is the number of differential metabolites, M is the number of metabolites annotated as a specific pathway, and m is the number of differential metabolites annotated as a specific pathway.

### Statistical analyses

Baseline sociodemographic and health-related characteristic analyses were performed using SPSS version 25.0 (SPSS Incorporation, Chicago, IL, USA), and *p* < 0.05 was regarded as statistically significant. Baseline sociodemographic and health-related characteristics were compared between the DC and the NDC using an independent t test for numeric variables and a chi-square test for categorical variables. Data with a normal distribution are expressed as the mean ± SD, and categorical variables are expressed as proportions.

## Results

### Characteristics of the study population

According to the exclusion criteria, we excluded 11 subjects; the remaining 368 were included in the experiment. Of the 368 subjects we included in the experiment, 49 were diagnosed with depression according to the diagnostic criteria as DC. The 319 people without depression were matched with 49 people according to age and sex as NDC. As shown in Table 1, there was no significant difference in sociodemographic lifestyle and healthy conditions between the DC and the NDC (*p* > 0.05). GDS scores (*p* < 0.001) was significantly different between the two groups.

**Table 1:** Baseline sociodemographic variables of the matched groups (N = 98).

Characteristic	DC (n = 49)	NDC (n = 49)	*p* Value
Age(years)	72.10 ± 5.12	73.47 ± 4.49	0.163
*Sex (%)*			0.671
Male	36.7	32.7	
Female	63.3	67.3	
*Smoking (%)*			0.727
No	91.8	89.8	
Yes	8.2	10.2	
*Drinking (%)*			0.133
No	85.7	73.5	
Yes	14.3	26.5	
BMI (kg/m^2^)	23.64 ± 3.59	24.27 ± 3.89	0.412
IPAQ (Met-min/wk)	5977.30 ± 5977.31	6385.78 ± 5391.08	0.712
Total cholesterol (mmol/L)	5.23 ± 0.94	5.29 ± 1.06	0.748
Triglycerides (mmol/L)	1.32 ± 0.70	1.27 ± 0.72	0.732
HDL (mmol/L)	1.41 ± 0.77	1.51 ± 0.39	0.129
LDL (mmol/L)	3.38 ± 0.78	3.39 ± 0.99	0.919
*Number of diseases*			
*Diebetes (%)*			0.316
No	75.5	83.7	
Yes	24.5	16.3	
*Hypertension (%)*			0.667
No	30.6	34.7	
Yes	69.4	65.3	
*Hyperlipidemia (%)*			0.505
No	87.8	91.8	
Yes	12.2	8.2	
*Stroke*			0.277
No	63.3	73.5	
Yes	36.7	26.5	
*Heart disease (%)*			0.671
No	63.3	73.5	
Yes	36.7	26.5	
MMSE	24.69 ± 4.67	23.94 ± 4.80	0.432
GDS score	14.71 ± 3.57	4.90 ± 2.37	< 0.001

*DC* depression cohort, *NDC* non-depression cohort, *BMI* body mass index, *IPAQ* international physical activity questionnaire, *HDL* high-density lipoprotein, *LDL* low-density lipoprotein, *MMSE* Mini-mental State Examination, *GDS score* Geriatric Depression Scale score.

### Untargeted GC/LC‒MS of samples

**Figure 2:**
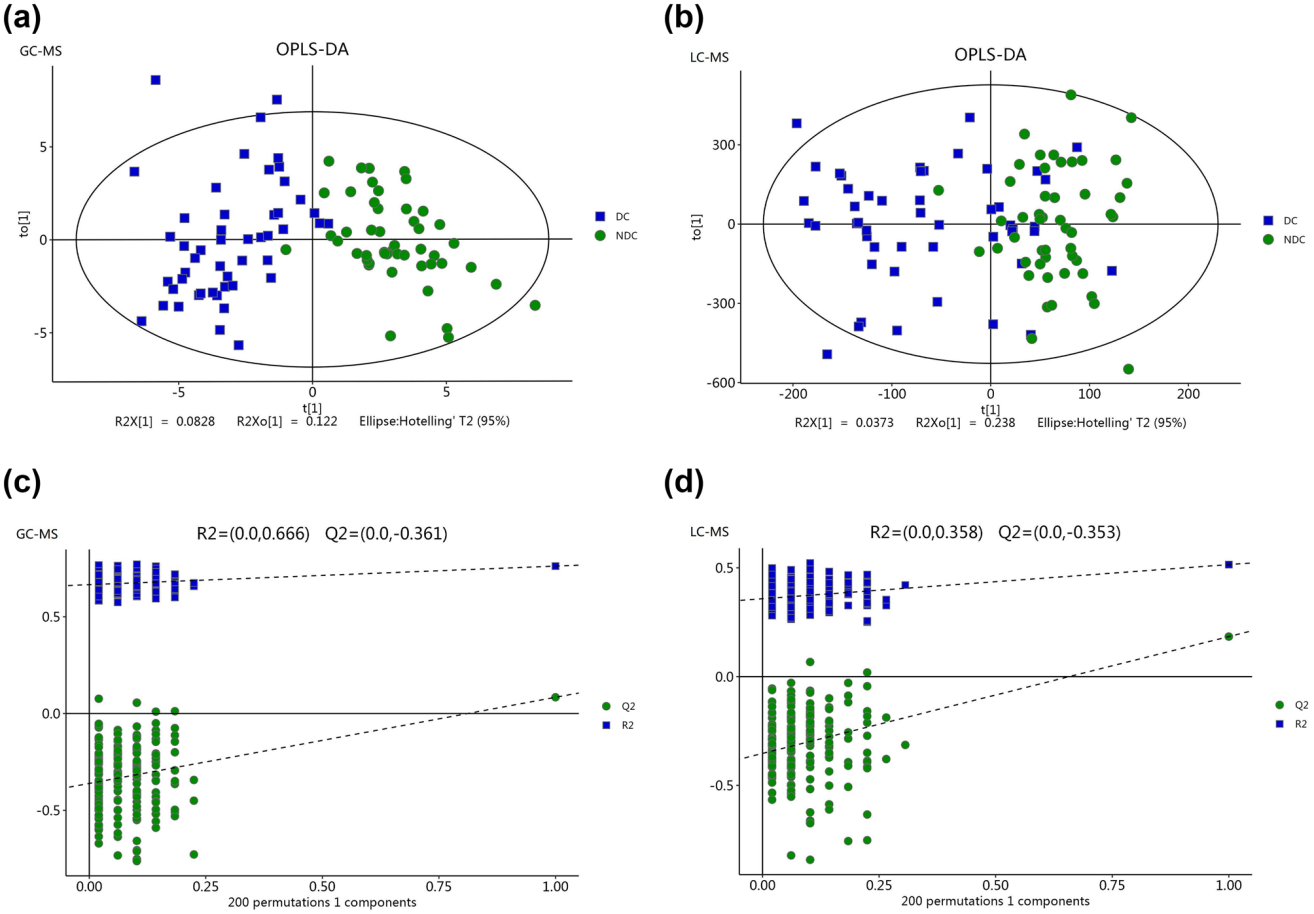
Multivariate date analysis of date from plasma between the depression cohert (DC) and non-depression corhort (NDC) base on GC/LC–MS. (**a**, **c**) OPLS-DA score plots (left panel) and statistical validation of the corresponding OPLS-DA model by permutation analysis (right panel) based on the GC–MS. (**b**, **d**) OPLS-DA score plots (left panel) and statistical validation of the corresponding OPLS-DA model by permutation analysis (right panel) based on the LC–MS. The two coordinate points are relatively far away on the score map, indicating that there is a significant difference between the two samples, and vice versa. The elliptical region represents a 95% confidence interval.

A total of 446 compounds were identified in plasma via GC‒MS, and 1012 were identified via LC‒MS. To determine the difference in plasma metabolites between the two groups of samples, we used the OPLS-DA model. The OPLS-DA model showed that there was obvious separation and little overlap between the two groups (Fig. 2A,B). Two hundred permutation tests were confirmed to not be overfitted (Fig. 2C,D).

### Potential biomarker analysis

**Figure 3:**
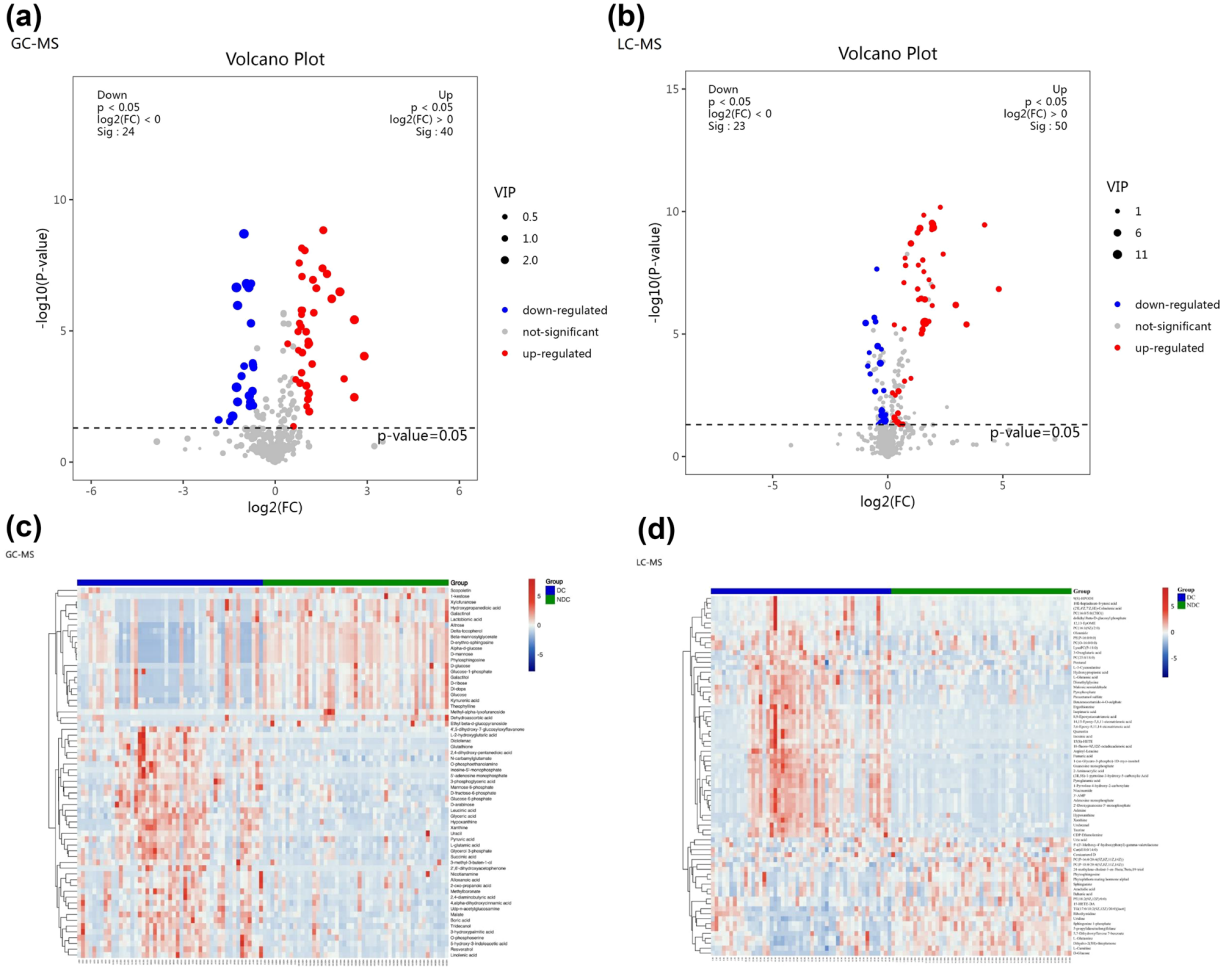
Volcano plot and hierarchical clustering based on the LC/GC–MS of serum metabolites obtained from the depression cohert (DC) and non-depression corhort (NDC). (**a**) Volcano plot based on GC–MS. (**b**) Volcano plot based on LC–MS. (**c**) Hierarchical clustering based on GC–MS. (**d**) Hierarchical Clustering based on LC–MS. In (**a**, **b**), the blue dot represents metabolite with a downward trend, red represents metabolites with an upward trend, and the gray origin represents that the change of metabolites is not obvious. The area size of the point is related to the VIP value. In (**c**, **d**), the color from blue to red illustrates that metabolites Hexpression abundance is low to high in hierarchical clustering.

**Figure 4:**
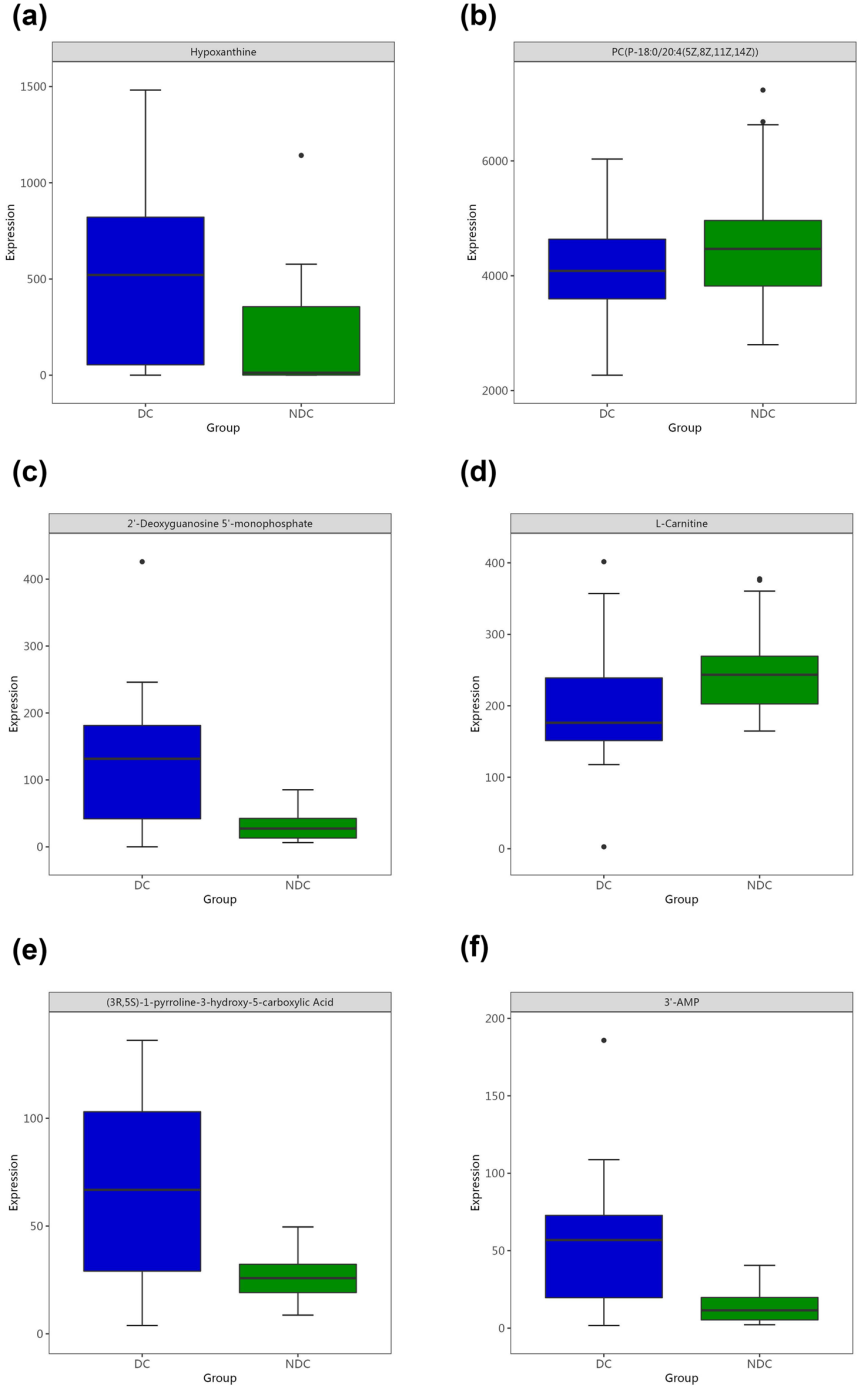

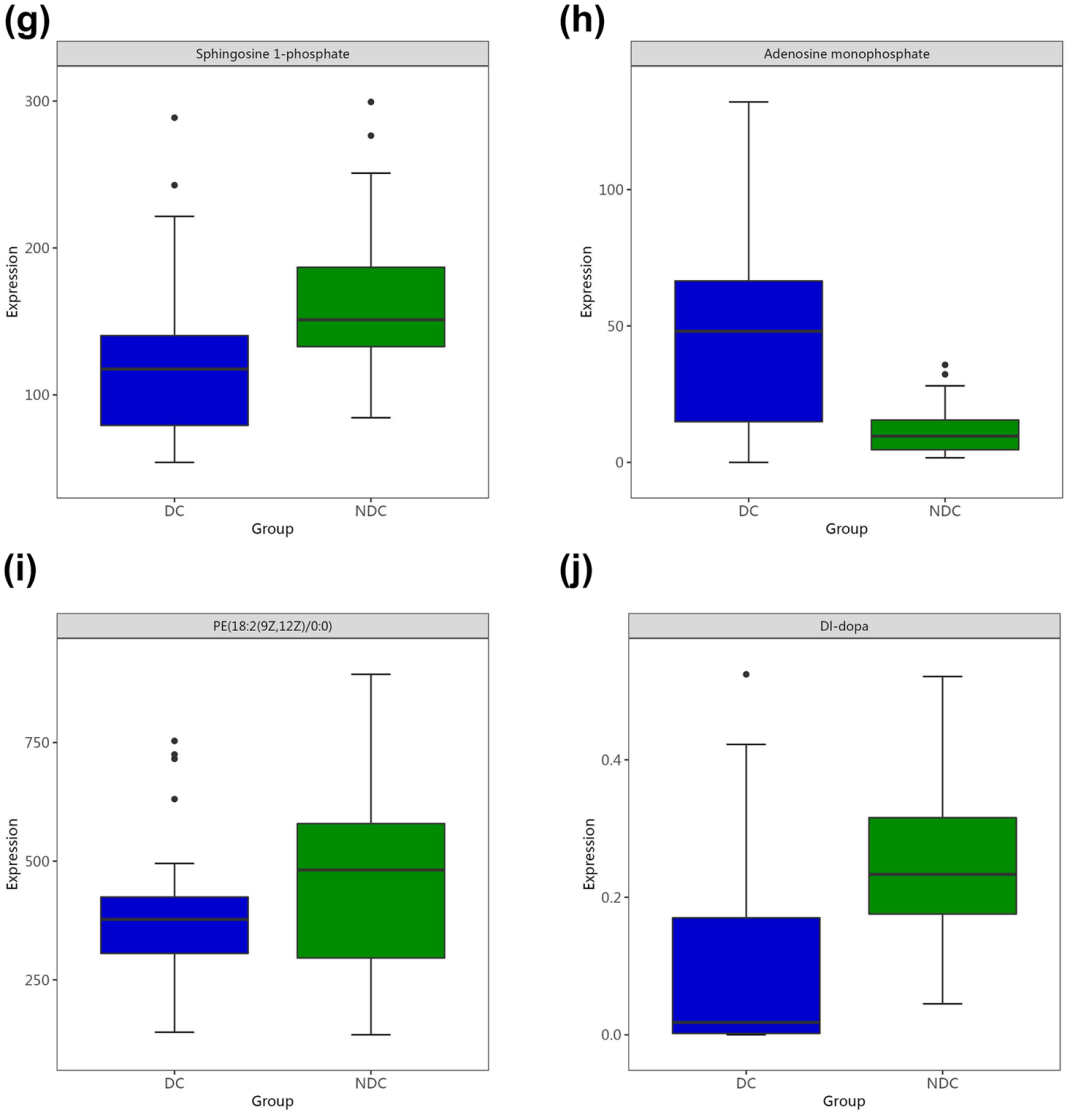
The top 10 metabolites are shown by box-and-whisker plots according to VIP values.

Among all identified metabolites, 64 metabolites identified via GC‒MS and 73 metabolites identified via by LC‒MS had significant contributions to the differentiation between the DC and the NDC, with VIP values > 1 and *p* values < 0.05 (Tables 2, 3). Three substances with the same name and KEGG ID were detected by both methods, including hypoxanthine, phytosphingosine, and xanthine. The volcanic map shows the P value and fold change value, thus proving the effectiveness of differential metabolites (Fig. 3A,B). Hierarchical clustering displayed the levels of these metabolites, in which colours represent higher levels (red) or lower levels (blue), with the intensity reflecting the corresponding concentration (Fig. 3C,D). The top 10 metabolites are shown by box-and-whisker plots according to VIP values (Fig. 4).

**Table 2:** Differential metabolites detected by GC–MS.

Metabolites	KEGG ID	^a^VIP value	^b^*p* Value	^c^FC	^d^TREND
Dl-dopa		3.82	< 0.001	0.42	↓
D-glucose	C00031	3.62	0.001	0.42	↓
Dehydroascorbic acid		3.55	0.018	0.38	↓
Altrose		3.53	< 0.001	0.49	↓
D-mannose	C00159	3.34	< 0.001	0.52	↓
Alpha-d-glucose	C00267	3.29	< 0.001	0.53	↓
Xylofuranose		3.15	0.003	0.56	↓
D-erythro-sphingosine		3.12	< 0.001	0.55	↓
Phytosphingosine	C12144	3.12	< 0.001	0.53	↓
Glucose		3.12	< 0.001	0.43	↓
Diclofenac	C01690	2.96	< 0.001	4.31	↑
Glutathione	C00051	2.91	< 0.001	5.98	↑
Scopoletin	C01752	2.90	0.005	0.43	↓
Hydroxypropanedioic acid		2.85	0.005	0.57	↓
Hypoxanthine	C00262	2.65	< 0.001	3.59	↑
Inosine-5′-monophosphate		2.65	< 0.001	7.48	↑
Glucose-1-phosphate		2.62	0.002	0.60	↓
L-2-hydroxyglutaric acid		2.53	0.003	5.96	↑
Beta-mannosylglycerate		2.43	< 0.001	0.58	↓
Kynurenic acid		2.40	< 0.001	0.60	↓
Galactinol		2.28	0.007	0.56	↓
5′-adenosine monophosphate		2.26	< 0.001	3.22	↑
Delta-tocopherol	C14151	2.25	< 0.001	0.58	↓
Alloxanoic acid		2.23	0.002	2.14	↑
Ethyl beta-d-glucopyranoside		2.19	0.001	0.47	↓
Uracil	C00106	2.07	0.012	2.16	↑
2-Oxo-propanoic acid		2.07	0.001	2.02	↑
Xanthine	C00385	2.00	< 0.001	2.96	↑
Malate	C00711	1.96	< 0.001	2.91	↑
Glycerol 3-phosphate		1.96	< 0.001	2.35	↑
Galactitol	C01697	1.90	< 0.001	0.60	↓
O-phosphoethanolamine		1.90	< 0.001	2.53	↑
Lactobionic acid		1.89	0.007	0.61	↓
3-Methyl-3-buten-1-ol		1.88	0.004	2.09	↑
Methyl-alpha-lyxofuranoside		1.87	0.025	0.28	↓
D-ribose	C00121	1.86	< 0.001	0.61	↓
Methylboronate		1.80	< 0.001	2.16	↑
4,Alpha-dihydroxycinnamic acid		1.79	< 0.001	1.85	↑
O-phosphoserine		1.76	< 0.001	2.11	↑
4′,5-Dihydroxy-7-Glucosyloxyflavanone		1.75	< 0.001	2.01	↑
Theophylline	C07130	1.75	< 0.001	0.50	↓
1-Kestose		1.73	0.028	0.36	↓
Resveratrol	C03582	1.70	< 0.001	2.39	↑
Linolenic acid		1.69	< 0.001	2.30	↑
Glyceric acid		1.66	< 0.001	1.95	↑
Pyruvic acid		1.62	< 0.001	1.82	↑
D-fructose-6-phosphate		1.57	< 0.001	1.79	↑
Boric acid	C12486	1.57	< 0.001	1.83	↑
3-Phosphoglyceric acid		1.53	< 0.001	2.10	↑
2,4-Diaminobutyric acid		1.53	0.001	1.75	↑
2′,6′-Dihydroxyacetophenone		1.52	0.001	4.74	↑
Mannose 6-phosphate		1.47	< 0.001	1.85	↑
5-Hydroxy-3-indoleacetic acid		1.47	< 0.001	1.80	↑
Succinic acid		1.47	< 0.001	1.72	↑
L-glutamic acid		1.46	< 0.001	1.82	↑
Tridecanol		1.40	< 0.001	1.68	↑
2,4-Dihydroxy-pentanedioic acid		1.39	< 0.001	1.73	↑
Udp-N-acetylglucosamine		1.30	0.001	1.76	↑
Leucinic acid		1.21	< 0.001	1.81	↑
N-carbamylglutamate		1.15	< 0.001	1.32	↑
Glucose 6-phosphate		1.14	0.001	1.59	↑
D-Arabinose	C00216	1.14	< 0.001	1.69	↑
3-Hydroxypalmitic acid		1.10	0.007	2.03	↑
Nicotianamine	C05324	1.09	0.043	1.51	↑

^a^Correlation coefficient and VIP value were obtained from OPLS-DA analysis.

^b^*p* Value determined from Student’s t-test.

^c^Fold change between depression cohort and non-depression cohort.

dRelative concentrations compared to non-depression cohort: ↑ = upregulated, ↓ = downregulated.

*FC* fold change, *VIP* variable importance for projection.

**Table 3:** Differential metabolites detected by LC–MS.

Metabolites	KEGG ID	^a^VIP	^b^*p* Value	^c^FC	^d^TREND
Hypoxanthine	C00262	12.06	< 0.001	3.05	↑
PC(P-18:0/20:4(5Z,8Z,11Z,14Z))		7.65	0.034	0.91	↓
2′-Deoxyguanosine 5'-Monophosphate	C00362	6.52	< 0.001	3.94	↑
L-Carnitine	C00318	4.68	< 0.001	0.80	↓
(3R,5S)-1-pyrroline-3-hydroxy-5-Carboxylic Acid	C04281	4.55	< 0.001	2.63	↑
3′-AMP	C01367	4.29	< 0.001	3.88	↑
Sphingosine 1-phosphate	C06124	3.93	< 0.001	0.74	↓
Adenosine monophosphate	C00020	3.89	< 0.001	3.79	↑
PE(18:2(9Z,12Z)/0:0)		3.85	0.014	0.83	↓
D-Glucose	C00221	3.71	< 0.001	0.51	↓
Quercetin	C00389	3.67	< 0.001	7.74	↑
15-HETE-DA		3.65	0.021	0.83	↓
Taurine	C00245	3.46	< 0.001	2.01	↑
Malonic semialdehyde	C00222	3.33	< 0.001	3.05	↑
9(S)-HPODE	C14827	3.30	0.039	1.33	↑
Pyroglutamic acid	C01879	3.15	< 0.001	2.60	↑
Paracetamol sulfate		3.11	< 0.001	2.85	↑
Uric acid	C00366	3.11	0.031	0.88	↓
12,13-EpOME	C14826	3.04	0.002	1.38	↑
Inosinic acid	C00130	3.03	< 0.001	10.69	↑
Phytosphingosine	C12144	2.83	0.002	0.68	↓
TG(17:0/18:2(9Z,12Z)/20:0)[iso6]		2.57	< 0.001	0.67	↓
Arginyl-Leucine		2.53	< 0.001	28.28	↑
Benzeneacetamide-4-O-sulphate		2.46	< 0.001	2.77	↑
1-Pyrroline-4-hydroxy-2-Carboxylate	C04282	2.44	< 0.001	2.45	↑
PC(25:0/18:0)		2.39	< 0.001	2.72	↑
10E-Heptadecen-8-ynoic acid		2.14	0.017	1.36	↑
L-3-Cyanoalanine	C02512	2.04	0.027	1.24	↑
5-propylideneisolongifolane		2.01	< 0.001	0.69	↓
PC(O-16:0/0:0)		1.85	0.027	1.21	↑
Pyrophosphate	C00013	1.76	< 0.001	2.46	↑
Ribothymidine		1.69	< 0.001	0.55	↓
Niacinamide	C00153	1.67	< 0.001	2.87	↑
L-Glutamic acid	C00025	1.67	< 0.001	1.71	↑
LysoPC(P-18:0)	C04230	1.66	0.025	1.21	↑
Sphinganine	C00836	1.63	0.039	0.81	↓
Guanosine monophosphate	C00144	1.58	< 0.001	18.43	↑
Arachidic acid	C06425	1.53	0.046	0.81	↓
L-Glutamine	C00064	1.52	0.002	0.89	↓
(2′E,4′Z,7′Z,8E)-Colnelenic acid	C16320	1.52	0.048	1.57	↑
Phytophthora mating hormone Alpha1		1.50	< 0.001	0.59	↓
Behenic acid	C08281	1.47	0.043	0.79	↓
Adenine	C00147	1.45	< 0.001	3.76	↑
Oleamide	C19670	1.44	0.001	1.65	↑
Uridine	C00299	1.40	< 0.001	0.72	↓
PC(16:0/5:0(CHO))		1.39	0.044	1.44	↑
Pentanal		1.38	0.003	1.15	↑
5′-(3′-Methoxy-4′-hydroxyphenyl)-gamma-valerolactone		1.35	0.049	0.75	↓
15(S)-HETE	C04742	1.35	< 0.001	2.86	↑
Ergothioneine	C05570	1.34	< 0.001	3.45	↑
24-Methylene-cholest-5-en-3beta,7beta,19-triol		1.25	0.012	0.83	↓
CDP-Ethanolamine	C00570	1.25	< 0.001	5.29	↑
Dimethylglycine	C01026	1.22	< 0.001	1.63	↑
Hydroxypropionic acid	C01013	1.22	< 0.001	1.22	↑
18-fluoro-9Z,12Z-octadecadienoic acid		1.18	< 0.001	2.52	↑
PC(P-16:0/20:4(5Z,8Z,11Z,14Z))		1.18	0.048	0.91	↓
Dolichyl beta-D-glucosyl Phosphate	C01246	1.16	0.050	1.42	↑
Undecanal		1.11	< 0.001	1.64	↑
Isopimaric acid	C09118	1.11	< 0.001	2.50	↑
Dihydro-2(3H)-thiophenone		1.11	< 0.001	0.82	↓
PC(16:1(9Z)/2:0)		1.10	0.001	2.01	↑
1-(sn-Glycero-3-phospho)-1D-Myo-inositol	C01225	1.10	< 0.001	4.87	↑
2-Aminoacrylic acid	C02218	1.09	< 0.001	2.95	↑
14,15-Epoxy-5,8,11-Eicosatrienoic acid	C14771	1.09	< 0.001	3.81	↑
Fumaric acid	C00122	1.08	< 0.001	1.68	↑
Cer(d18:0/14:0)		1.07	0.020	0.94	↓
3-Oxoglutaric acid		1.07	0.003	1.25	↑
PE(P-16:0/0:0)		1.05	0.043	1.20	↑
Xanthine	C00385	1.03	< 0.001	2.96	↑
5,7-Dihydroxyflavone 7-benzoate		1.03	< 0.001	0.57	↓
8,9-Epoxyeicosatrienoic acid	C14769	1.03	< 0.001	3.89	↑
5,6-Epoxy-8,11,14-eicosatrienoic acid	C14768	1.03	< 0.001	3.46	↑
Conicasterol D		1.03	0.049	1.16	↑

^a^Correlation coefficient and VIP value were obtained from OPLS-DA analysis.

^b^*p* value determined from Student’s t-test.

^c^Fold change between depression cohort and non-depression cohort.

dRelative concentrations compared to non-depression cohort: ↑ = upregulated, ↓ = downregulated.

*PC* phosphatidylcholine, *LysoPC* lysophosphatidylcholine, *PE* phosphatidylethanolamine, *TG* triglyceride, *Cer* ceramide, *FC* fold change, *VIP* variable importance for projection.

### Evaluation of the metabolite panel and for the diagnosis of depression

ROC curve analysis further evaluated the diagnostic performance of differential metabolites. There were 11 metabolites with AUC values > 0.8, including 1-(sn-glycero-3-phospho)-1D-myo-inositol (AUC = 0.842, 95% CI 0.752–0.932), ergothioneine (AUC = 0.834, 95% CI 0.753–0.915), taurine (AUC = 0.832, 95% CI 0.745–0.919), 15(S)-HETE (AUC = 0.824, 95% CI 0.739–0.909), guanosine monophosphate (AUC = 0.821, 95% CI 0.733–0.909), quercetin (AUC = 0.814, 95% CI 0.719–0.908), 14,15-epoxy-5,8,11-eicosatrienoic acid (AUC = 0.810, 95% CI 0.718–0.903), diclofenac (AUC = 0.809, 95% CI 0.722–0.897), 3'-AMP (AUC = 0.808, 95% CI 0.711–0.904), CDP-ethanolamine (AUC = 0.805, 95% CI 0.709–0.901), and inosine-5′-monophosphate (AUC = 0.804. 95% CI 0.709–0.899) (Table 4).

**Table 4:** Metabolites with AUC greater than 0.8.

Metabolites	AUC	Specificity	Sensitivity	Cutoff	95% CI
1-(sn-Glycero-3-phospho)-1D-yo-inositol	0.842	0.796	0.898	1.054	0.752–0.932
Ergothioneine	0.834	0.612	0.939	3.632	0.753–0.915
Taurine	0.832	0.694	0.939	41.978	0.745–0.919
15(S)-HETE	0.824	0.653	0.959	5.243	0.739–0.909
Guanosine monophosphate	0.821	0.673	1.000	2.024	0.733–0.909
Quercetin	0.814	0.714	0.939	10.049	0.719–0.908
14,15-Epoxy-5,8,11-eicosatrienoic acid	0.810	0.673	0.959	2.026	0.718–0.903
Diclofenac	0.809	0.673	0.857	0.001	0.722–0.897
3′-AMP	0.808	0.673	0.959	31.111	0.711–0.904
CDP-Ethanolamine	0.805	0.673	0.959	2.610	0.709–0.901
Inosine-5′-monophosphate	0.804	0.673	0.959	0.001	0.709–0.899

### Metabolic pathways change depression

**Figure 5:**
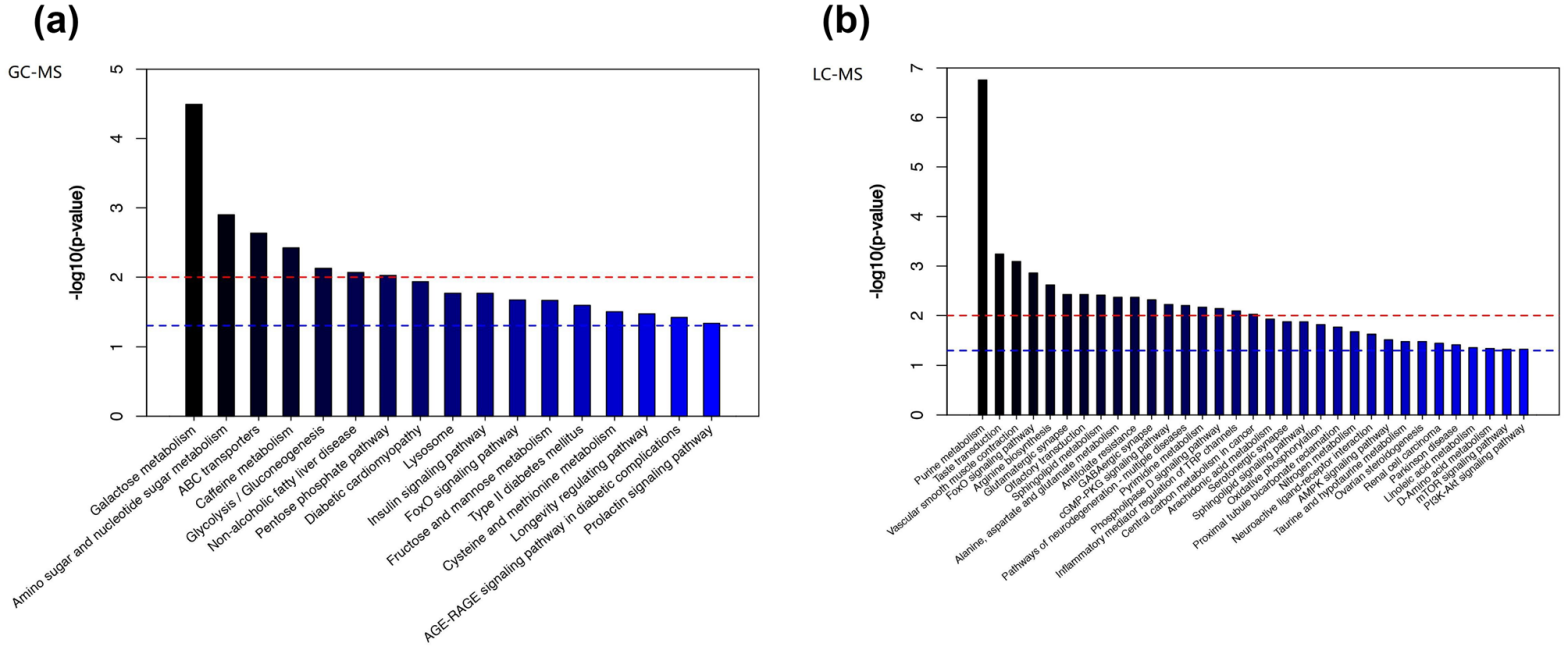
Metabolic pathway analysis based on the differentiated plasma metabolites. (**a**) Metabolic pathway analysis based on GC–MS. (**b**) Metabolic pathway analysis based on LC–MS.

To understand which metabolic pathways may affect depression, we conducted metabolic pathway enrichment (Fig. 5). We found that these metabolites are mostly related to purine metabolism and galactose metabolism.

## Discussion

To our knowledge, our study is the first to use a nontargeted metabolomic method to study the plasma metabolic profile of depression in Chinese community-dwelling older adults. A total of 1458 metabolites were detected by LC‒MS and GC‒MS, including 137 different metabolites with VIP values > 1 and *p* values < 0.05. To identify reliable biomarkers, we made a volcano map, and we performed hierarchical clustering, box diagram analysis and ROC curve analysis for different metabolites. Furthermore, we also enriched the metabolic pathways and analysed the affected metabolic pathways.

In a previous study of major depressive disorder, 822 metabolites were detected in plasma using LC, and 17 metabolic pathway changes were found^22^. Thirty-seven metabolites were detected by GC‒MS in the plasma of pregnant women with antenatal depressive symptoms^23^. Compared to using only LC–MS or GC‒MS, we detected more metabolites using both LC–MS and GC‒MS and discovered more differential metabolites and changes in metabolic pathways^12–14,22,23^. Previous studies have found changes in amino acid, fatty acid, and purine metabolism in plasma samples of depression, and our study also found similar findings. In addition, we also found changes in FoxO signaling and Ampk signaling pathway pathways, which are involved in cellular autophagy^24,25^. Therefore, our results suggest that depression may be related to cellular autophagy.

Our results revealed that 1-(sn-glycero-3-phospho)-1D-myo-inositol had a high diagnostic value. 1-(sn-Glycero-3-phospho)-1D-myo-inositol, also known as glycerophosphoinositol, is produced through membrane phosphatidylinositol through two successive deacylation steps catalysed by phospholipase A2IVα^26,27^. According to a study, high glycerophosphoinositol levels indicate cellular phenomena associated with the activation of RAS/mitogen-activated protein kinase (MAPK) pathways^27^. Our metabolic pathway enrichment results showed that depression was closely associated with the MAPK signaling pathway. Ras/MAPK pathway alterations play a critical role in human brain structure and white matter microstructure^28^. In a study of depression in elderly individuals, it was found that white matter changes in elderly individuals with depression and that the changed white matter was related to cognitive control and emotional regulation^29^. Therefore, 1-(sn-glycero-3-phospho)-1D-myo-inositol may affect the structure of white matter through the Ras/MAPK pathway, leading to depression.

In this study, we found significant changes in several metabolic pathways, the most important of which was the purine metabolic pathway. Compared with the non-depression cohort, the depression cohort was characterized by higher levels of purine compounds (2′-deoxyguanosine 5′-monophosphate, 3′-AMP, adenosine monophosphate, xanthine, guanosine monophosphate, inosinic acid, adenine, and hypoxanthine) and lower levels of uric acid. Purine compounds are the substrate of purine metabolism, and uric acid is the end product of purine metabolism^30^. Thus, based on these findings, we suggest that downregulated purine metabolism may occur in older adults with depression. A previous study also showed that uric acid in the plasma of patients with depression decreased^30^. Uric acid has an important role in vivo as an antioxidant that provides more than 60% antioxidant activity in plasma^31,32^. Depression is associated with increasing levels of oxidative stress^33^. Excessive oxidative stress leads to damage to the brain function of patients and various psychiatric symptoms. Downregulation of purine metabolism and lack of sufficient uric acid to fight oxidative stress result in brain damage and depression. However, purine metabolism was upregulated in a metabonomic study of children and adolescents with major depressive disorder^12^. Therefore, the role of purine metabolism in depression needs further study.

We have made certain achievements in metabolomics research on depression. Three substances (hypoxanthine, phytosphingosine, and xanthine) were detected by LC‒MS and GC‒MS. In addition, many articles also mentioned these metabolites^9,22,34^, so our results are reliable and repeatable. Hypoxanthine and xanthine affect the occurrence of depression through purine metabolism (described above). Phytosphingosine is classified as a sphingolipid, and the D-erythro-sphingosine that we detected is a sphingolipid^35^. There is a large amount of sphingolipids in the central nervous system. Their metabolites are an important structure of biological membranes and participate in many cell signal transduction pathways as second messengers^35,36^. Sphingolipids are acylated to produce ceramide^37^. A study injected ceramide into the hippocampus of mice, and then the proliferation, maturation, and survival of neurons in mice decreased, leading to depressive behaviour^38^. However, our results showed that the concentration of ceramide in plasma decreased in the depression cohort. This may be because ceramide enters the central nervous system through the blood‒brain barrier and accumulates in the hippocampus, resulting in depression and a decrease in ceramide concentration in the periphery^39^.

However, our research still has some limitations. First, our sample size is small, including only elderly individuals aged 65 and above in Chongming, Shanghai. Secondly, our article only compared the metabolite differences between the two groups without an in-depth study of the metabolites. The Hamilton Depression Rating Scale is the most common tool for clinically diagnosing depression, but the GDC scale was used in this study. The two scales may have some differences in the diagnosis of depression. In future studies, we will increase the sample size and pay attention to the differences between the two scales for diagnosing depression to identify better biomarkers. At the same time, we will also conduct some in-depth studies on metabolites in subsequent studies, and we will also validate the metabolites in the depression model again.

## Conclusion

In conclusion, our results suggest that there are several plasma metabolites associated with depression. Several of these metabolites have high diagnostic value and may be used as markers for depression diagnosis. Through further study of differential metabolites, we can also find changes in the metabolic pathway of depression.

## Data Availability

The datasets generated during and/or analysed during the current study are not publicly available due to protect study participant privacy but are available from the corresponding author on reasonable request.
